# Antioxidant Capacity of Proteins and Hydrolysates from the Liver of Newborn Piglets, and Their Inhibitory Effects on Steatosis *in vitro*

**DOI:** 10.17113/ftb.58.04.20.6657

**Published:** 2020-12

**Authors:** Ruilin Zhang, Lasheng Yin, Jian Chen, Xuewu Zhang

**Affiliations:** College of Food Science and Engineering, South China University of Technology, Wshan Road 381, 510640 Guangzhou, PR China

**Keywords:** newborn piglet liver, protein hydrolysates, antioxidant activity, oleic acid, lipid accumulation

## Abstract

**Research background:**

Non-alcoholic steatohepatitis is a potentially progressive hepatic disorder that can lead to end-stage liver disease and hepatocellular carcinoma. The inhibitory effects of proteins and hydrolysates from the liver of newborn piglets on hepatic steatosis in oleic acid-induced hepatocellular carcinoma (HepG2) cells were investigated *in vitro*.

**Experimental approach:**

The extracted proteins from the liver of newborn piglets were hydrolysed with papain, pepsin, trypsin and Alcalase. Based on the comparison of different enzyme digestion conditions, a protein hydrolysis protocol was established to obtain hydrolysates with lipid-lowering effect.

**Results and conclusions:**

Trypsin-digested liver protein hydrolysate from newborn piglets exhibited strong antioxidant activity and good inhibitory effects against lipogenesis and cholesterol accumulation in HepG2 cells at the concentration of 150 μg/mL, with a triglyceride decrease of (43±3) % and cholesterol decrease of (31±5) %, compared with model group induced with 0.75 mM oleic acid. The addition of trypsin-digested liver protein hydrolysate from newborn piglets (300 μg/mL) decreased alanine aminotransferase and aspartate aminotransferase activities and increased superoxide dismutase activity.

**Novelty and scientific contribution:**

This study demonstrated that the trypsin-digested liver protein hydrolysate from newborn piglets has a potential preventive effect against non-alcoholic fatty liver disease in its early stage, and a potential use as the modulator of lipid overaccumulation in form of food supplements.

## INTRODUCTION

Non-alcoholic fatty liver disease (NAFLD) is caused by the lipid accumulation in the liver without intaking excessive amounts of alcohol. NAFLD develops from a simple steatosis with the absence of inflammation and non-alcoholic steatohepatitis and can progress to liver cirrhosis and malignant hepatic cancer (*1*, *2*). Non-alcoholic steatohepatitis is characterized by accumulation of fat, inflammation and injury of liver cells (*2*). A prevailing theory of NAFLD pathogenesis, known as the ’double-hit’ hypothesis, involves both inflammation and oxidative stress (*3*). Generally, lipid overaccumulation is caused by an imbalance between the uptake (anabolism) and output (catabolism), which causes oxidative stress and triggers the overproduction of reactive oxygen species (ROS). As a significant pathway in activating the ’second hit’, oxidative stress has an important role in hepatic cell damage and dysfunction. There is a subsequent vicious cycle of lipid overaccumulation in the fatty liver, resulting in hepatocyte injury and inflammation (*3*, *4*). In addition, liver injury may induce mitochondrial dysfunction and peroxidation damage, causing steatosis. In turn, oxidative stress decrease brings a balance between the production of ROS and antioxidant defences, enhancing lipid metabolism and lowering adipose accumulation effectively (*4*). Thus, the reduction of oxidative stress can indirectly regulate fatty acid synthesis and control fatty acid degradation occurring in NAFLD patients.

Animal products such as bioactive proteins and protein hydrolysates have potent liver regeneration effects and may stimulate the regeneration of the liver after various types of liver injuries. These products include milk protein hydrolysate (*5*), tuna dark muscle protein (*6*), and pork liver protein hydrolysate (*7*). Additionally, animal liver has long been recognized as a source of several nutritional proteins, bioactive polypeptides such as hepatocyte growth factor (HGF) and hepatocyte stimulator substance (*8*). HGF is reported as a multifunctional cellular growth factor that can be found in a wide variety of life cells and tissues, which plays a pivotal role in the liver regeneration (*9*, *10*). Moreover, hepatic stimulator substance and hepatic growth factor regulate the symptoms of NAFLD and convey resistance to hepatic injury by protecting mitochondrial function (*7*, *11*).

The objective of our work is to study the inhibitory effects of liver proteins and their hydrolysates of newborn piglets on hepatic steatosis in oleic acid-induced HepG2 cells. In this study, to evaluate their lipid-lowering capacity, we investigated triglyceride (TG) and cholesterol accumulation; the activities of intracellular biochemical enzymes, alanine aminotransferase (ALT), aspartate aminotransferase (AST) and superoxide dismutase (SOD); cellular antioxidant activity and the inhibition of lipid peroxidation.

## MATERIALS AND METHODS

### Material

TSK gel G2000 SWXL and Sephadex G50 (50-150 μm) were obtained from Sigma-Aldrich Corp., Merck, St. Louis, MO, USA. Pepsin (300 000 U/g), papain (800 000 U/g), trypsin (1:250 U/g), cholesterol, oleic acid, and bicinchoninic acid protein assay kit were obtained from Guangzhou Qiyun Biotech Co., Ltd., Guangzhou, PR China. Alcalase 2.4 L (2.4 U/g) and 3-(4,5)-dimethylthiahiazo (-z-y1)-3,5-di-phenytetrazoliumromide (MTT) were from Sigma-Aldrich Corp., Merck. Dulbecco's modified eagle medium (DMEM), fetal bovine serum, bovine serum albumin (BSA), pravastatin, the alanine aminotransferase (ALT), aspartate aminotransferase (AST) and superoxide dismutase (SOD) testing kits were purchased from Nanjing Jiancheng Bioengineering Institute, Nanjing, PR China. All other reagents were of analytical grade.

### Preparation of proteins from the liver of newborn piglets

The liver from a newborn pig (5 weeks old) was obtained from a market in Shenzhen City, Guangdong Province, PR China. The total protein content of the liver was 19.8%. The liver was cut into pieces and washed under running tap water, then drained. First, the drained liver pieces were dissolved in water at a liver to water ratio of 1:2.8 (*m*/*V*). After homogenization, the liver solution was frozen at −20 °C for 6 h and ultrasonicated with 1200DT ultrasonic cell crusher (Biosafer Technologies Co., Ltd., Beijing, PR China) at a power of 300 W for 15 min. We repeated these processes five times. The soluble fraction (extracted liver proteins from newborn piglet (LPNP) were centrifuged using Allegra™ 25R centrifuge (Beckman Coulter, Inc., Bremerhaven, Germany) at 8694×*g* for 10 min followed by filtration. The supernatants were lyophilized by FDU-1200 lyophilizer (Tokyo Rikakikai Co., Ltd., Tokyo, Japan) and stored at −20 °C.

### Protein hydrolysis and optimization

The extracted LPNP were hydrolysed with four different proteases (pepsin, papain, trypsin and Alcalase). To achieve this, the LPNP were dissolved in water (*m*/*V*=1:3), and the pH, temperature and reaction time were adjusted to the optimal conditions for each protease. Following the hydrolysis, the enzymes were inactivated by heating at 100 °C for 15 min. The pepsin-, papain-, trypsin- and Alcalase-digested liver protein hydrolysates from newborn piglets (LPH-pepsin, LPH-papain, LPH-trypsin and LPH-Alcalase, respectively) were obtained by centrifugation at 869×g (Allegra™ 25R centrifuge (Beckman Coulter, Inc., Brea, CA, USA) for 10 min, followed by filtration. The supernatants were lyophilized and stored at −20 °C. The triglyceride (TG) inhibitory activity of each enzymatic hydrolysate (LPH-pepsin, -papain, -trypsin and -Alcalase) on oleic acid-induced HepG2 cells *in vitro* was measured.

The protein hydrolysis with the highest activity (LPH-trypsin) was selected for optimization. After the initial single-factor experiments, based on the Box-Behnken principle, a response surface design for three variables (time, temperature and pH) was used to optimize the LPNP hydrolysis processes, and TG inhibitory activity was used as the response variable (Table S1). The supernatants were obtained by centrifugation at 8694×*g* for 10 min, then lyophilized and stored at −20 °C.

### Determination of antioxidant activities of newborn piglet liver proteins and their hydrolysates

Oxygen radical absorbance capacity (ORAC) assay was demonstrated according to Qiu *et al.* (*12*). Each of newborn piglet LPH-pepsin, LPH-papain, LPH-trypsin and LPH-Alcalase was mixed with phosphate buffer (75 mM, pH=7.4) to concentration of 1 mg/mL. A volume of 20 μL aliquot of each sample (LPNP and LPH) was mixed with 120 μL fluorescein (*c*=300 nM) in each well. Plates were then incubated at 37 °C for about 30 min in a microtiter plate reader (R164720 Millipore CytoFluor; Millipore Corporation, Bedford, MA, USA). A solution of 2,2-azobis (2-amidinopropane) dihydrochloride (AAPH, *c*=80 mM, *V*=50 μL; Sigma-Aldrich Corp., Merck, St. Louis, MO, USA) was added to each well; the plate was incubated under constant shaking, and the fluorescence was measured immediately in a microtiter plate reader (R164720 Millipore CytoFluor, Millipore Corporation), at 1-minute intervals for 60 min. To evaluate the absorbance, the fluorescence was measured at 485 and 530 nm. The ORAC value was expressed as Trolox equivalents using linear regression in the concentration range of 6.25-50 μM (*13*).

The radical scavenging activity of DPPH was demonstrated according to a previously described method (*14*) with slight modification. Briefly, a solution of 0.16 mM DPPH (Nanjing Jiancheng Bioengineering Institute) was prepared in absolute methanol. Then, 180 μL of DPPH solution were mixed with 20 μL of the samples (LPNP and LPH) in 96-well plates, which were incubated at room temperature for 30 min, then shaken at 500 rpm thoroughly. The absorbance was read at 517 nm using a microplate spectrophotometer system (model 550; Bio-Rad, Hercules, CA, USA). The control group plates contained the same volume of DPPH without any extract.

### Molecular mass distributions

The molecular mass distributions of LPNP and LPHs were evaluated using HPLC (model LC-10ATvp pump and DGU-12A degasser; Thermo Fisher Scientific, Germering, Germany). During the experiment, samples were introduced to the gel column (TSK gel G2000 SWXL; Tosoh Biosciences, Grove City, OH, USA), and the absorbance was monitored at *λ*=220 nm. The flow phase contained phosphate buffer (pH=7.0), and the flow velocity was 0.5 mL/min.

### Cytotoxicity of newborn piglet liver proteins and their hydrolysates

HepG2 cells were purchased from the Animal Experimental Center of Sun Yat-Sen University, Guangzhou, PR China. These cells were seeded into a 96-well plate (2∙10^4^ cells/well). The cells were incubated for 24 h in an incubator (model MCO-20AIC; Sanyo Electric Biomedical Co., Osaka, Japan) at 5% CO_2_ and 37 °C. Then, the medium was removed, and each well was washed once with phosphate-buffered saline (PBS). The medium was replaced with serum-free DMEM containing different concentrations of LPNP and LPHs, and the cultures were incubated for 24 h. The cells were washed twice with PBS. Then, 200 μL of 0.5 mg/mL MTT was added to each well and incubated for 4 h. Then, the MTT solution was removed, and 150 mL dimethyl sulfoxide were added. The absorbance was read at 490 nm using a microplate reader (Thermo Fisher Scientific, Waltham, MA, USA) and cell viability was calculated as follows:

FTB-58-455-e1.eps

### Inhibitory effect on oleic acid-induced fatty liver model in vitro

HepG2 cells were incubated for 24 h in a 6-well plate at a density of 2∙10^5^ cells/well. The regular medium was removed from the wells, and they were washed once with PBS. The cells were incubated with a medium containing 0.75 mM bovine serum albumin (BSA) with oleic acid for another 24 h. Newborn piglet LPNP and LPH were respectively added to the 6-well plate and incubated for 24 h. Cells cultured in media with only 1% BSA or 100 mg/L pravastatin were used as the control and positive sample, respectively.

### Measurement of intracellular triglyceride concentration and oil red O staining

The intracellular TG concentration was measured using triglyceride assay kit (Beijing BHKT Clinical Reagent Co., Ltd. Beijing, PR China), and the total protein concentration were measured with a bicinchoninic acid protein assay kit. Intracellular TG concentration were expressed in micrograms of TG per milligram of cellular protein. Oil red O staining was applied to measure the degree of preadipocyte differentiation according to a previous study (*15*).

### Measurement of intracellular cholesterol levels

After 24 h of cultivation, the HepG2 cells were harvested and lysed, then a 2:1 chlorine/methanol solution was added. After being left for 12 h, the cholesterol content of the liquid was measured. We used qualitative and quantitative chromatographic analysis to analyse cholesterol mass fractions. Intracellular compounds were investigated with an HPLC system with a diode array detector (model SPD-M10Avp; Shimadzu, Kyoto, Japan). The solvent methanol was used for the mobile phase. The cytochylema from each group was injected into the column using a 20-μL loop valve. The flow rate was set to 1.0 mL/min, and absorbance was measured at 210 nm using UV spectrophotometer (model SPD-M10Avp; Shimadzu). Components were tentatively identified by comparing their retention times (*t*_R_) with the authentic standards at 210 nm and cholesterol mass fraction was calculated as follows:

FTB-58-455-e2.eps

where *A_t_*_=12.5_ min is the peak area of the sample at *t*_R_=12.5 min.

### Measurement of intracellular SOD, ALT and AST activities

SOD, ALT and AST activities were measured using enzymatic kits according to the manufacturer's instructions (Nanjing Jiancheng Bioengineering Institute). The total protein levels were tested with a bicinchoninic acid assay kit.

### Statistical analysis

Data were presented as mean value±standard deviation of triple replicates. The significance of the differences (p<0.05, p<0.01) between the two groups was assessed using the paired *t*-test. All statistical analyses were performed using SPSS (*16*).

## RESULTS AND DISCUSSION

### Cytotoxicity of newborn piglet liver proteins and their hydrolysates, and their effects on cell proliferation and TG accumulation in oleic acid-induced hepatic steatosis

**Fig. 1:**
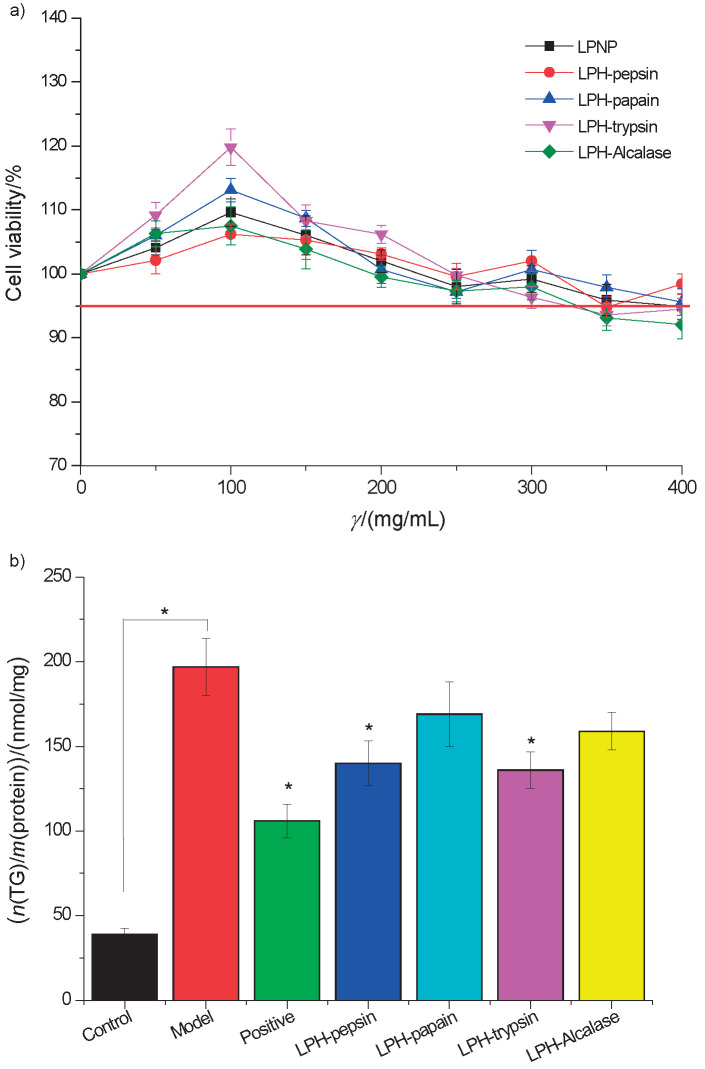
The results show: a) cytotoxic effect of newborn piglet liver proteins and their hydrolysates on HepG2 cells. Cell viability was determined by the 3-(4,5-dimethylthiazol-2-yl)-2,5-diphenyl tetrazolium bromide (MTT) assay and is expressed as a percentage of control cells with sample-free DMEM medium, b) effect of exposure to 0.75 mmol/L oleic acid on intracellular triglyceride (TG) accumulation and inhibitory effect of newborn piglet liver proteins and their hydrolysates at *γ*=300 μg/mL on TG overaccumulation in HepG2 cells. *p<0.05 relative to the model (0.75 mM oleic acid-induced cell). All tests were performed in triplicate, and the results are presented as mean value±S.D. LPNP=liver proteins of newborn piglet, LPH-pepsin=pepsin-digested PLP hydrolysate, PLH-trypsin=trypsin-digested LPNP hydrolysate, LPH-Alcalase=Alcalase-digested LPNP hydrolysate, BSA=bovine serum albumin

In this study, through a combination of freeze-thawing and ultrasonication, 91.9% (*m*/*m*) of crude proteins were extracted from newborn piglet liver. Different concentrations of LPNP, LPH-pepsin, LPH-papain, LPH-trypsin and LPH-Alcalase were incubated with HepG2 cells to determine their cytotoxicity (Fig. 1a). The results show that the five samples did not significantly reduce the cell viability over a period of 24 h at 300 μg/mL (p>0.05). Safe doses of 50-300 μg/mL were used to determine TG accumulation in the HepG2 cells. The data indicated that LPH-trypsin was the most efficient suppressor of TG accumulation in the hepatocyte (Fig. 1b), with a maximum TG decrease of 40.3% at 150 μg/mL. LPH-pepsin and LPNP caused the maximum TG decrease of 30.7 and 32.2%, respectively (Fig. 1b). TG decrease rate of LPH-trypsin was lower than that of the pravastatin group (100 mg/L, 51.2%). According to the literature, the hepatic TG clearance capacity of LPH-trypsin is weaker than of bioactive small molecules (polyphenols and flavonoids) (*14*). Liu *et al.* (*15*) suggested that blueberry phenolic acid is efficient in inhibiting TG accumulation in HepG2 cells, and maximal TG decrease was 58.6%, when the concentration was 100 μg/mL. Alshammari *et al.* (*17*) showed that nimbolide from *Azadirachta indica* successfully reduces intracellular cholesterol by 47.3% relative to the control. Nevertheless, relative to other protein or polypeptides, LPH-trypsin has a strong capacity of decreasing hepatic TG accumulation.

### Protein hydrolysis optimization

**Fig. 2:**
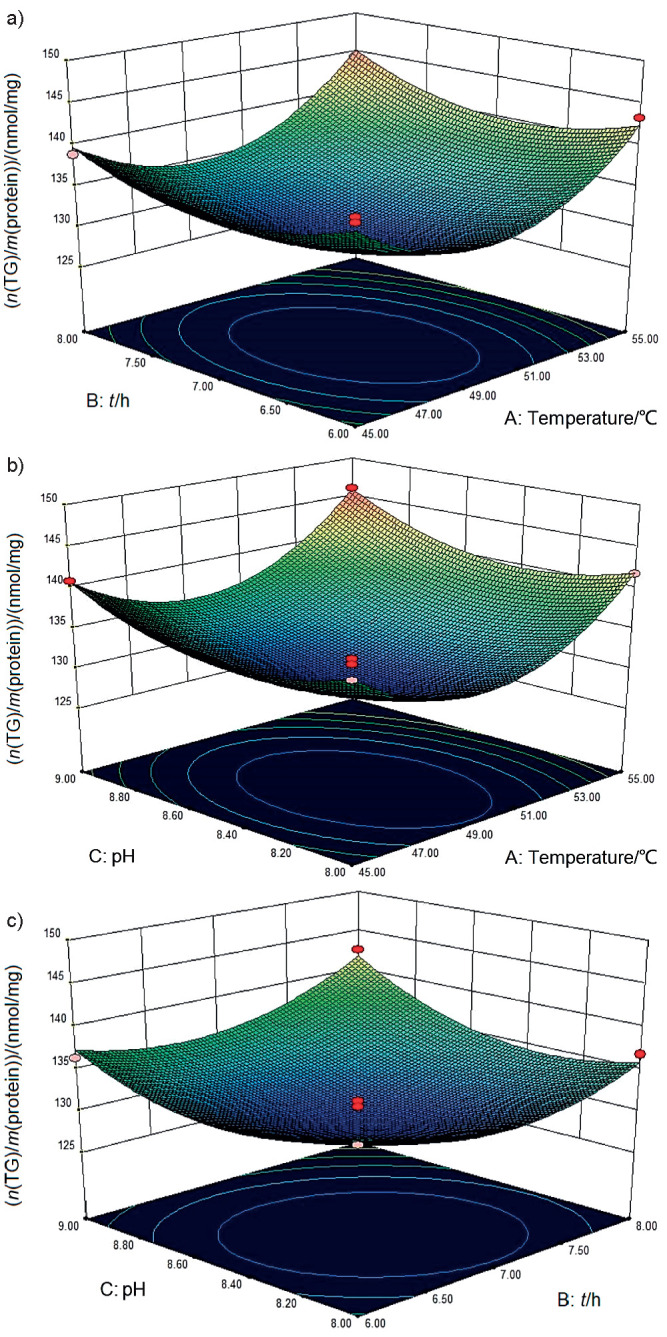
Effects of cross-interaction among the three parameters on the degree of hydrolysis of trypsin: a) pH values and temperatures, b) pH values and enzyme-to-substrate ratio, and c) temperature and enzyme-to-substrate ratio

Trypsin hydrolysis was conducted on the extracted proteins. The single-factor experiment indicated that the optimum conditions for trypsin hydrolysis included a pH=8.5, a temperature of 50 °C and a reaction time of 7 h. Subsequently, a response surface design was performed with the three variables, temperature, pH and the reaction time, using TG concentration (in nmol per mg of protein) as the objective function (Fig. 2). Using regression analysis, we obtained the following response equation:

FTB-58-455-e3.eps

According to the sum of squares presented in Table 1, the contribution of the three factors to the degree of hydrolysis can be placed in the following order: temperature>pH>reaction time. The optimal hydrolysis conditions (minimum TG accumulation) were determined to be a pH=8.4, temperature of 49.27 °C and reaction time of 6.91 h. Experimental verification under the conditions of pH=8.5, temperature of 49 °C, and a reaction time of 7 h determined the TG concentration in protein to be 129.8 nmol/mg, close to the theoretical value of 128.8 nmol/mg. The lipid-lowering ability of the optimized hydrolysates was roughly improved by 3.2%, compared with unoptimized LPH-trypsin (40.3%, *γ*=150 μg/mL).

**Table 1:** Analysis of variance (ANOVA) of the liver proteins from newborn piglets hydrolysed with trypsin

Parameter	Sum of squares	df	Mean square	F value	p-value
Model	475.9101	9	52.8789	43.66345	<0.0001
temperature/°C (A)	41.67845	1	41.67845	34.41495	0.0006
*t*/h (B)	7.860613	1	7.860613	6.490707	0.0382
pH (C)	23.83951	1	23.83951	19.68489	0.0030
AB	0.6561	1	0.6561	0.541758	0.4856
AC	0.3364	1	0.3364	0.277774	0.6144
BC	5.784025	1	5.784025	4.776016	0.0651
A^2^	238.5346	1	238.5346	196.9641	<0.0001
B^2^	58.7142	1	58.7142	48.48181	0.0002
C^2^	62.71078	1	62.71078	51.78188	0.0002
Residual	8.477395	7	1.211056		
Lack of fit	6.328275	3	2.109425	3.926119	0.1098
Pure error	2.14912	4	0.53728		
Cor total	484.3875	16			

p<0.05 significant, p<0.01 very significant

### Molecular mass distribution of newborn piglet liver proteins and their hydrolysates

As shown in Table 2, the fraction of peptides in LPH-trypsin with molecular mass<3000 Da was up to 85.07%, which was the highest among the LPNP and LPHs, with the peptides in the molecular mass range <1000 and 1000-3000 Da being 38.14 and 46.93%, respectively. The fraction of peptides in newborn piglet LPNP with molecular mass<3000 Da was 65.51%, with the peptides of <1000 Da at 39.29% and 1000-3000 Da at 26.22%. Additionally, the fraction of large peptides (>5000 Da) of LPNP was up to 20.7%. Combined with previous results, low molecular mass peptides of LPH-trypsin may contribute to its antioxidant and lipid lowering abilities. Moreover, other reports have also proposed a small molecular peptide with good bioactivity (*18*, *19*).

**Table 2:** Molecular mass distributions of piglet liver proteins and their pepsin and trypsin hydrolysates

*M*/Da	Peak area/%		
LPNP	LPH-pepsin	LPH-trypsin
>10000	13.352	3.953	1.162
10000-5000	7.343	4.945	1.503
5000-3000	13.479	11.379	12.26
3000-1000	39.29	42.292	46.93
<1000	26.22	37.02	38.14

LPNP=liver proteins from newborn piglets, LPH-pepsin=pepsin-digested LPNP hydrolysate, LPH-trypsin=trypsin-digested LPNP hydrolysate

### Antioxidant activities of newborn piglet liver proteins and their hydrolysates

Newborn piglet LPH-trypsin exhibited higher radical scavenging activity than the other hydrolysates. DPPH radical scavenging capacity of LPH-trypsin (IC_50_=7.2 mg/mL) was higher than of LPH-pepsin (IC_50_=8.2 mg/mL) and LPNP (IC_50_=9.2 mg/mL) (data not shown). The investigation of the oxygen radical absorbance capacity (ORAC), expressed as Trolox equivalents, showed that the samples (LPNP, LPH-pepsin, and LPH-trypsin) had different capacities. LPH-trypsin had the highest ORAC activity ((594±2) μM/g) among the hydrolysates. LPH-pepsin produced the second-highest ORAC activity ((427±2) μM/g), whereas LPNP had the lowest ((254±4) μM/g) (p<0.05). LPH-trypsin showed significantly (p<0.05) higher reducing power than the other extracts (data not shown). These results suggest that LPH-trypsin has a strong oxygen radical scavenging (ROS) activity, which might contribute to its lipid-lowering effect. Domínguez-Pérez *et al.* (*20*) showed that hepatocyte growth factor reduces free cholesterol-mediated lipotoxicity by inhibiting overproduction of ROS, compared with normal cells. Xie *et al.* (*21*) also pointed out that dihydromyricetin reduces oleic acid-induced lipid accumulation in L02 and HepG2 cells by inhibiting lipogenesis and oxidative stress. Vidyashankar *et al.* (*22*) reported that quercetin effectively ameliorates NAFLD symptoms by decreasing triacyl glycerol accumulation and increasing cellular antioxidants in oleic acid-induced hepatic steatosis in HepG2 cells.

### Inhibitory effects of the liver proteins and their hydrolysates on fat accumulation in steatotic HepG2 cells

**Fig. 3:**
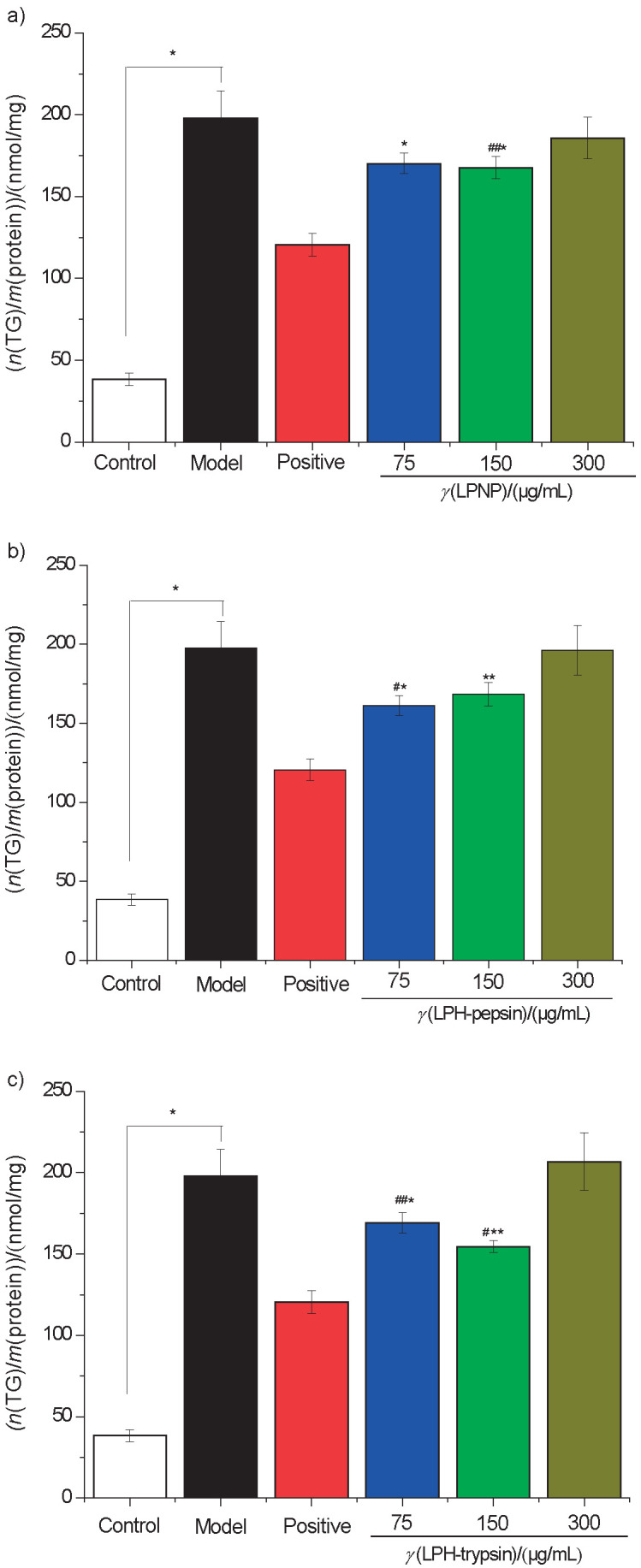
Effect of exposure to 0.75 mmol/L oleic acid on intracellular triglyceride (TG) accumulation. Inhibitory effect of: a) LPNP, b) LPH-pepsin, and c) LPH-trypsin at 75, 150 and 300 μg/mL on TG overaccumulation in HepG2 cells; #p<0.05 and ##p<0.01 relative to the control sample, *p<0.05 and **p<0.01 relative to the model sample. Abbreviations as in Fig. 1

**Fig. 4:**
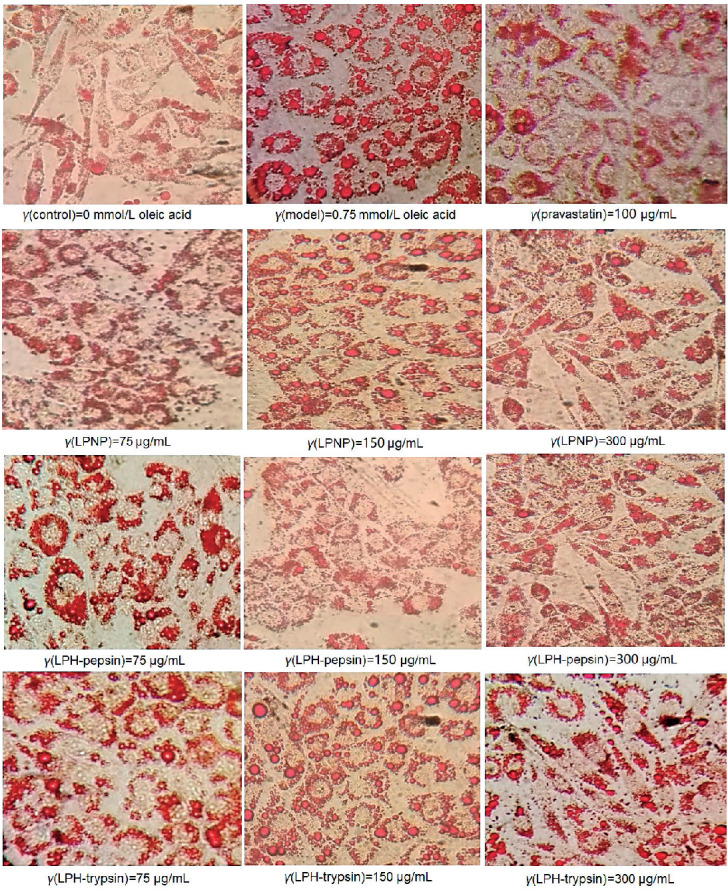
Oil red O staining of corresponding fractions (LPNP, LPH-pepsin and LPH-trypsin) at 75, 150 and 300 μg/mL, indicating triglyceride (TG) overaccumulation in HepG2 cells. Original magnification was 200×, scale bars represent 75 μm. Abbreviations as in Fig. 1

In HepG2 cells, the effectiveness of LPNP, LPH-pepsin and LPH-trypsin in inhibiting TG and fat overaccumulation was evaluated (Fig. 3). The results showed that LPNP and LPH-pepsin showed the maximum TG decrease rate of roughly 30.7 and 32.21%, respectively (Fig. 3a and Fig. 3b). LPH-trypsin was effective in suppressing TG accumulation in steatotic cells, with a maximum TG decrease rate of 43.3% at a concentration of 150 μg/mL (Fig. 3c), which was weaker than blueberry phenolic acid (58.6%, *γ*=100 μg/mL) (*15*). To investigate whether newborn piglet liver proteins and their hydrolysates affect oleic acid-induced cellular steatosis, a simple qualitative method to analyse the amount of stored intracellular lipid droplets was performed using oil red O staining. The cells in the normal group exhibited irregular polygons, clear edges and fewer red-stained lipid droplets (Fig. 4). After treatment with 0.75 mM oleic acid, HepG2 cells began to appear circular and intracellular lipid droplets became larger with a deeper red colour. The number and size of the lipid droplets significantly reduced after the treatment with newborn piglet LPNP and LPH, relative to those of the model group. At concentration of 150 μg/mL, the intracellular lipid droplets of cells treated with LPH-trypsin became smaller with a slightly less intense red colour. In concurrence with the TG accumulation data, LPH-trypsin showed significantly most effective inhibition of TG (p<0.05) in HepG2 cells at concentration of 150 μg/mL.

### Effects of newborn piglet liver proteins and their hydrolysates on cholesterol secretion in HepG2 cells

**Fig. 5:**
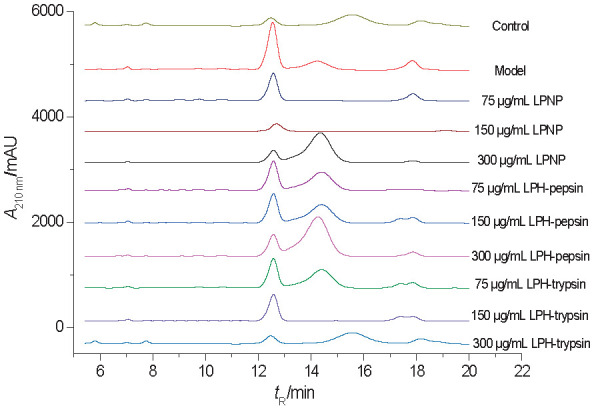
HPLC analysis of the effect of exposure to 0.75 mmol/L oleic acid and treatment with LPNP, LPH-pepsin and LPH-trypsin on intracellular cholesterol accumulation in HepG2 cells. Pure cholesterol used as standard with a peak at 210 nm after *t*_R_=12.5 min. Abbreviations as in Fig. 1

Cholesterol peaks were observed at absorbance of 210 nm and *t*_rR_=12.5 min. As shown in Fig. 5, the amount of cholesterol significantly decreased in HepG2 cells treated with liver proteins and their hydrolysates compared with the model group. LPH-trypsin was the most efficient at suppressing cholesterol accumulation, with a maximum clearance rate of 30.5% at a concentration of 150 μg/mL.

### Effects of newborn piglet liver proteins and their hydrolysates on SOD in HepG2 cells

**Fig. 6:**
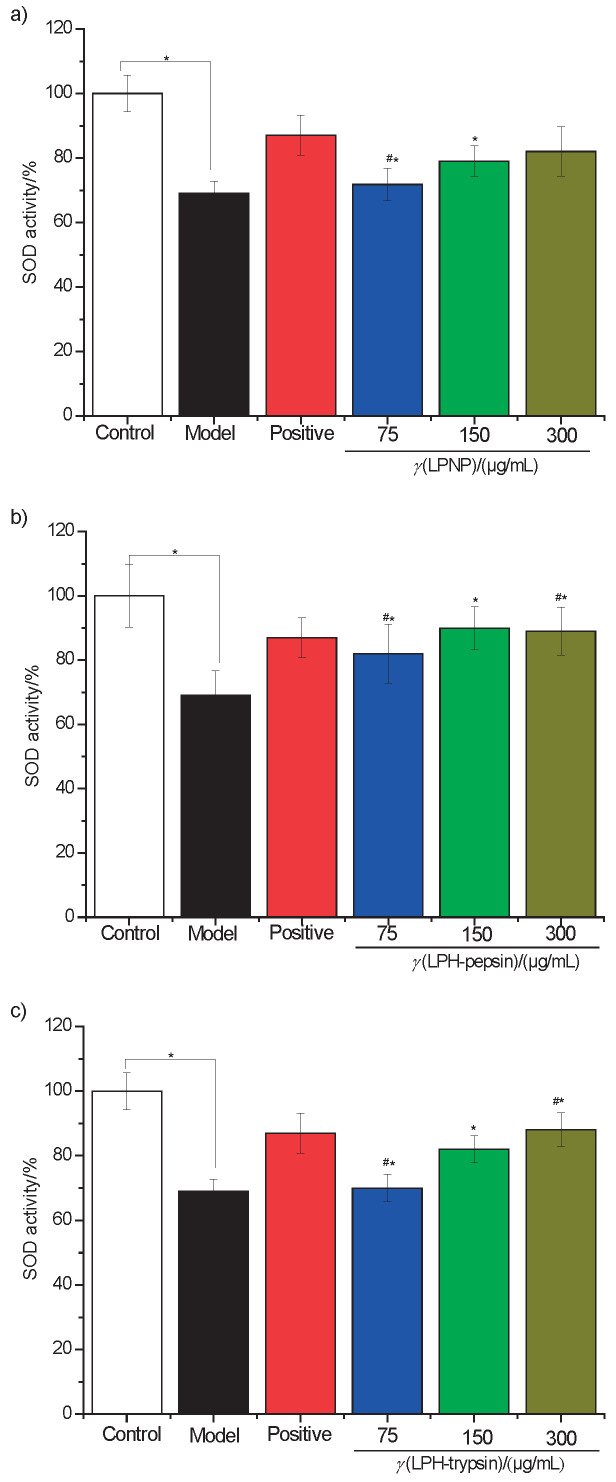
Effect of exposure to 0.75 mmol/L oleic acid on the intracellular antioxidant enzyme superoxide dismutase (SOD) in fatty cells and inhibitory effect of: a) LPNP, b) LPH-pepsin and c) LPH-trypsin on SOD activities in HepG2 cells; #p<0.05 relative to the control sample; *p<0.05 relative to the model sample. Abbreviations as in Fig. 1

Superoxide dismutase (SOD) was measured in the cell to investigate whether the lipid-decreasing effect of liver proteins and their hydrolysates *in vitro* is related to their antioxidant capacity. Fig. 6 shows that the total SOD activity was inhibited in the cells treated with 0.75 mM oleic acid compared with the activity in the control group. However, SOD activity increased after the treatment with newborn piglet liver proteins and their hydrolysates. According to the obtained results, SOD activity significantly increased by 23.53% (p<0.05) after treatment with 300 μg/mL LPH-trypsin (Fig. 6c), compared with the model cells.

### Effects of newborn piglet liver proteins and their hydrolysates on the production of ALT and AST in HepG2 cells

In general, high ALT and AST levels in the blood are indicators of hepatitis. Specifically, ALT and AST are indicators of hepatic cell inflammation. Therefore, in the cell model, ALT and AST release assays were used to evaluate the progress of NAFLD. Oleic acid increases AST and ALT levels in steatotic hepatic cell significantly, compared with those in the control group (p<0.05), suggesting that HepG2 cells treated with oleic acid exhibited oxidative stress. After the treatment with LPH-trypsin, ALT and AST activities reduced significantly (23 and 15.2%, respectively), relative to the model group (data not shown). There is no significant difference between the AST and ALT activities in the groups treated with 300 μg/mL LPH-trypsin and in the control group. These results show that the biological properties of LPH-trypsin increased the levels of AST and ALT activity at 150-300 μg/mL in HepG2 cells (p<0.05). Previous literature reported that bioactive molecules effectively reduce fat accumulation in hepatocytes by renewing AST and ALT activities (*7*-*9*). Yin *et al.* (*23*) showed that Hugan Qingzhi tablets exert a preventive effect against hepatic steatosis by enhancing AST and ALT enzyme activities. Shimiz*u et al.* (*7*) showed that consumption of pork liver protein hydrolysate decreases the body mass and inhibits hepatic lipogenesis in rats significantly. The mechanisms of the liver protein or protein hydrolysate inhibition of TG synthesis in hepatocytes and its relationship to their antioxidant properties remain to be elucidated.

To study the lipid-lowering effects and antioxidant activities of newborn piglet liver proteins and their hydrolysates using *in vitro* experiments, HepG2 cells were incubated with a mixture of oleic acid to induce cellular steatosis. The number of lipid droplets and the levels of TG and cholesterol increased significantly in the cells treated with 0.75 mM oleic acid, which indicates that the steatosis model *in vitro* had been established successfully. Obviously, 0.75 mM oleic acid causes oxidative stress in hepatocytes, and it has been well documented that hepatic antioxidant systems are significantly decreased in several chronic liver diseases (*20*, *22*). Newborn piglet liver proteins and their hydrolysates reduced the size of lipid droplets, the intracellular TG, and cholesterol content significantly. The involvement of classical intracellular ROS scavengers, such as SOD, is of fundamental importance in the development of therapeutical approaches to oxidation-based liver pathologies. It is known that SOD plays an important role in hepatocyte defence against ROS, free radicals and electrophilic metabolites (*23*-*25*). Therefore, the present data showed that newborn piglet liver proteins and their hydrolysates protect hepatocytes against oxidative stress and ameliorate intracellular TG, cholesterol and lipid overaccumulation. Compared with oleic acid-induced model, LPH-trypsin caused a decrease of 43.3% TG in oleic acid-induced HepG2 cells at 150 mg/mL, which was little lower than that of hypolipidaemic drug pravastatin group (*γ*=100 mg/L, 51.2%). The capacity of LPH-trypsin to decrease TG was slightly lower than of small bioactive molecules (polyphenols, flavonoids and phenolic acids) (*14*, *15*), and higher than in other types of protein hydrolysate (*5*, *7*). These results indicate that LPH-trypsin inhibits well the TG accumulation in HepG2 cells.

Literature demonstrated that NAFLD is a multifactorial disease resulting from a complex interaction of environment, enzymes and metabolism (*3*, *23*, *26*). The protective effects of bioactive molecules are induced against oleic acid-induced hepatic steatosis by regulation of intracellular AST and ALT *in vitro* (*23*). Moreover, the experimental results show that AST and ALT activities significantly decreased in the LPH-trypsin group. The data indicated that 300 μg/mL LPH-trypsin possess a strong hepatoprotective effect because aminotransferase is generally considered to be an indicator of hepatocyte injury. Hence, LPH-trypsin reduced the progression of hepatic steatosis because of its antioxidant properties, which can protect mitochondria from peroxidation damage, inhibit TG overaccumulation and recover normal metabolism.

Oleic acid can increase lipid, TG and cholesterol accumulation and induce mitochondrial dysfunction, which increase steatosis in the cells. Newborn piglet liver proteins and their hydrolysates reduced these symptoms, exerting inhibitory effects against the overaccumulation of TG in hepatic cells. In addition, liver proteins and hydrolysates effectively weaken intracellular oxidative stress at high concentrations, while ineffectively preventing TG overaccumulation (*26*, *27*). Newborn piglet liver proteins and their hydrolysates are a mixture of hepatocyte growth factor, hepatocyte stimulator substance and various free amino acids, which confer resistance to hepatic injury and oxidative stress by improving mitochondrial functions.

However, the lipid-decreasing mechanisms of LPH-trypsin require further investigation. We aim to focus in the future on the cellular energy regulation in NAFLD and signalling pathways of the relative lipogenic enzymes. Experiments using model animals are needed to confirm the present findings, especially the lipid-decreasing effects of LPH-trypsin in the alimentary tract and liver.

## CONCLUSIONS

The results of the study clearly suggested that the liver proteins and their hydrolysates of newborn piglets have the potential to scavenge free radicals and prevent hepatic steatosis. The triglyceride (TG) inhibitory capability was different in the proteins and their hydrolysates. Generally, the proteins and all of the hydrolysates showed good inhibitory activity against triglyceride and cholesterol accumulation, and trypsin-digested hydrolysate was the most effective lipid accumulation inhibitor with a maximum TG reduction of 43.3%. Therefore, liver proteins and their hydrolysates of newborn piglets exert good protective effects to reduce the risk of non-alcoholic fatty liver disease.

## Figures and Tables

**Table S1:** Response surface design of variance for liver proteins from newborn piglets hydrolysed with trypsin

Group	Temperature/°C (A)	*t*/h (B)	pH (C)	(*n*(TG)/*m*(protein))/(nmol/mg)
1	45	7	9	140.9
2	45	7	8	137.8
3	50	7	8.5	129.4
4	50	7	8.5	131.3
5	55	7	8	141.8
6	45	6	8.5	139.5
7	55	6	8.5	143.3
8	45	8	8.5	138.9
9	55	7	9	146.0
10	55	8	8.5	144.3
11	50	8	8	136.9
12	50	7	8.5	130.5
13	50	6	9	136.4
14	50	6	8	135.5
15	50	8	9	142.6
16	50	7	8.5	129.8
17	50	7	8.5	130.2

TG=triglyceride
